# Supplementary material for: Reliability and validity of the PORTRAIT-10 tool for assessing complex health care needs in French-speaking people living with chronic pain
Source: PLoS One. 2025 Oct 29;20(10):e0335734. doi: 10.1371/journal.pone.0335734 (PMC12571328; doi:10.1371/journal.pone.0335734)
Supplement: S1 File — (DOCX) [file pone.0335734.s001.docx]

LINK TO PORTRAIT-10

<https://www.portrait-10.ca/>
