# Supplementary material for: Reliability and validity of the PORTRAIT-10 tool for assessing complex health care needs in French-speaking people living with chronic pain
Source: PLoS One. 2025 Oct 29;20(10):e0335734. doi: 10.1371/journal.pone.0335734 (PMC12571328; doi:10.1371/journal.pone.0335734)
Supplement: Attachment — Submitted filename: Plos one review-16-02_25.docx [file pone.0335734.s003.docx]

This is an assessment of PORTRAIT-10, a newly developed questionnaire for complex medical needs with the INTERMED-Self Assessment (IMSA), the Pain Catastrophizing Scale (PCS), and the Pain Self-Efficacy Questionnaire (PSEQ) in a French speaking population.

The manuscript is rigorous and well written. It seems to give a thorough analysis of the practical aspects of the questionnaire. I like the way that they have presented their analysis. It is important to note that I am not an expert in this methodology employed but, it seems to follow logical, from what I can see, likely best practice guidelines.

I do not have any general concerns about the article. It might be useful to specify that the article is relevant to the French speaking population in the title.

In the abstract on line 48, it might be nice to see the characteristics (age/pain duration, sex) specific to the respondent population only included - is this 199?

In the introduction, the authors may wish to include that this type of assessment would also have some utility in assessments for patients in trial recruitment, which is key as therapeutic interventions for patients with chronic pain are limited.

Lines 71-75 convey an important message well.

If there is any more information available on the study population this might be useful to understand the generalisability of the analysis.

Perhaps, a little more information on any real-world evidence of improving healthcare outcomes by understanding the health complexity in this way.

Do the authors have any idea why the attrition rate was high, although there are still good numbers of respondents.
